# Lipedema and Hypermobility Spectrum Disorders Sharing Pathophysiology: A Cross-Sectional Observational Study

**DOI:** 10.3390/jcm14207195

**Published:** 2025-10-12

**Authors:** Elettra Fiengo, Andrea Sbarbati

**Affiliations:** 1 Studio Iris, Pomezia, 00071 Rome, Italy; 2 Department of Neurological, Biomedical and Movement Sciences, Section of Anatomy and Histology, University of Verona, 37134 Verona, Italy

**Keywords:** lymphedema, joint pathology, connective tissue, adipose tissue, fibrosis

## Abstract

**Background/Objectives:** Lipedema is a chronic, progressive disorder of the adipo-fascial tissue characterized by abnormal subcutaneous fat accumulation, inflammation, fibrosis, pain, and edema. Despite its considerable impact on patients’ quality of life, it remains underdiagnosed. Recent studies have suggested a potential overlap between lipedema and hypermobility spectrum disorders (HSDs), both involving connective tissue dysfunction. This work explores the shared pathophysiological features of lipedema and HSD, highlighting clinical correlations, comorbidities, and the need for integrated diagnostic and therapeutic approaches. **Methods**: A cross-sectional observational study was conducted through an online survey targeting individuals with lipedema and a control group with lymphedema. The questionnaire assessed symptoms typically associated with HSD, including musculoskeletal, gastrointestinal, urogynecological, vascular, and neuropsychological manifestations. Descriptive statistics were used to evaluate clinical patterns in both groups. **Results**: Among the lipedema patients, 44% reported joint hypermobility and 60% recalled being hypermobile during childhood. High rates of pediatric overweight (50%), low muscle tone (55%), and exercise-induced fatigue (70%) were observed. Adult symptoms included joint pain (notably in the ankles, knees, cervical spine, sacrum, and feet), digestive issues (50%), and thyroid disorders (24.4%). Compared with the control group, patients with lipedema showed significantly more connective tissue-related motor deficits and systemic symptoms. **Conclusions**: Connective tissue laxity may play a critical role in the pathogenesis of lipedema, contributing to multisystemic manifestations through vascular, lymphatic, gastrointestinal, and musculoskeletal involvement. The high prevalence of HSD-like features calls for a paradigm shift in the understanding of lipedema as a systemic disorder. Early identification of connective tissue alterations, especially in children with familial predisposition, could enable timely interventions, potentially mitigating disease progression. A multidisciplinary, evidence-based approach is essential for accurate diagnosis and effective management.

## 1. Introduction

Lipedema is a multifactorial, chronic, and underdiagnosed disease characterized by adipose tissue proliferation, edema, inflammation, and fibrosis.

Pain, reduced motor function, and comorbidities impair the patients’ quality of life, affecting both physical and mental health, especially in advanced stages. Although the etiopathogenesis is not clear, there appears to be an interaction between genetic, hormonal, vascular, and inflammatory factors. It is believed that lipedema genes are inherited in an autosomal dominant pattern with sex limitation [1]. Evidence shows a role of estrogens in adipose tissue dysregulation [2], and the presence of higher extracellular fluid has been detected by bioelectrical impedance analysis (BIA) in women with lipedema compared with controls [3], alongside stage-progressive interstitial fibrosis and a high percentage of macrophages [4]. Endothelial alterations have been found in biopsies of subcutaneous tissue mainly in affected areas but also in unaffected regions, suggesting a global involvement of the entire adipose organ [5].

Many studies have described the presence of hypermobile joints, suggesting a connective tissue disorder associated with increased compliance and/or loss of tissue elasticity. Connective tissue is an integral part of the structure of blood and lymphatic vessels and influences vessel compliance, resulting in increased permeability, especially under higher pressure [6]. Systemic structural alterations of connective tissue in lipedema could explain many of the symptoms reported by patients and described in the literature among the disease’s comorbidities.

Lymphedema is a chronic, progressive and often disabling condition characterized by the accumulation of protein-rich interstitial fluid due to congenital or acquired lymphatic dysfunction. It should be regarded as a clinical syndrome encompassing tissue fluid accumulation, adipose tissue deposition, and fibrosis with secondary skin changes occurring in advanced stages. Early recognition and staging are essential to prevent irreversible structural damage and functional impairment. When lymphatic drainage falls below the functional load, protein-rich interstitial fluid accumulates, triggering sterile inflammation and progressive fibro-adipose tissue remodeling [7,8]. Lymphedema is classified as primary (due to congenital or hereditary lymphatic malformations) or secondary (after oncologic surgery and/or radiotherapy, infections such as filariasis, trauma, or severe obesity) [7,8]. Staging ranges from Stage 0 (latent) to Stage III (elephantiasis). Differential diagnosis is essential to distinguish lymphedema from lipedema and other causes of chronic limb swelling such as venous insufficiency or systemic edema. Lymphedema typically shows distal involvement (including the dorsum of the foot and toes) may be asymmetric, with pitting in the early stages: Stemmer’s signs (the inability to pinch the skin at the base of the second toe or finger) is usually positive, reflecting dermal fibrosis [7].

“Ehlers-Danlos syndromes (EDSs) are a group of inherited connective tissue disorders caused by abnormalities in the structure, production and/or processing of collagen”. Joint hypermobility is a common feature of many subtypes of EDSs and other inherited connective tissue diseases and is defined as the ability of a joint to move “beyond normal limits along physiological axes”, may involve few or many joints, and may be entirely asymptomatic. For patients with symptomatic joint hypermobility who do not meet either the new hypermobile Ehlers-Danlos syndrome (hEDS) criteria or another specific condition, the 2017 classification introduced hypermobility spectrum disorders (HSDs) [9]. hEDSs and HSDs present a complex range of signs and symptoms of varying degrees and combinations that make these conditions difficult to recognize. The diagnosis of hEDSs, to date, requires evidence of generalized joint hypermobility and two or more of: (A) systemic manifestations of generalized hypermobility-type connective tissue disorder (HCTD); (B) positive family history of hEDS; and (C) evidence of musculoskeletal complications [10].

A recent global survey with more than 3900 participants provided an epidemiological profile of individuals with hEDSs, HSDs, and relative differences between the two groups, revealing a surprisingly high burden of multisystem comorbidities, profound diagnostic delays, and significant unmet clinical needs. Chronic pain, the most recurrent symptom (98.8% hEDS and 92.7 HDS) with an average of six painful joints per participant, musculoskeletal complications such as migraine, scoliosis, Raynaud’s syndrome, nerve entrapments, and herniated disc. Fatigue, dizziness, and brain fog are the most reported autonomic disorders along with heart palpitations and thermoregulatory dysfunction, suggesting significant autonomic involvement. Irritable bowel syndrome, reflux, dysmotility are among the most common gastrointestinal symptoms with abdominal pain, nausea, bloating, and constipation. Pelvic pain, irregular menstruation, pain during sexual intercourse, endometriosis, PCOS, complications during pregnancy, and recurrent urinary tract infections and incontinence are among the most frequent disorders in the pelvic area. Atopic dermatitis, stretch marks, urticaria, and mast cell activation syndrome are among the allergic and immunological conditions as well as astigmatism, myopia, hyperopia, macular degeneration, keratoconus, retinal detachment, sensitivity to light, visual disturbances, dry eyes, double vision, temporomandibular joint disorders, dental crowding, high or narrow palate, and frequent caries. Anxiety, depression, insomnia, attention deficit hyperactivity disorder, autism, and obsessive compulsive disorder are the manifestations regarding mental health and sleep [11]. Triggering events such as environmental, pubertal, or viral factors are often reported close to the onset of symptoms [12] in early adolescence, with patients reporting injury, gastrointestinal disturbances, and aches often mistaken for growing pains [13,14].

Several studies have described the involvement of connective tissue in lipedema, highlighting the prevalence of: hypermobility in 50% of patients with fluid leakage from vessels and capillary dysfunction [15], reduced elasticity of skin and aorta [16], easy bruising [17], and systemic symptoms including fatigue, pain, diffuse swelling, altered gait, joint hypermobility, and fatigue [18].

Increased compliance of connective tissue occurs in the presence of excess interstitial fluid originating from dysfunctional blood vessels with fluid leakage into the interstitial space and among dermal fibers of the skin [6]. Varicose veins, arthritis, and hypothyroidism are described among common comorbidities [19].

In patients with lipedema, upregulated microRNAs have been identified that are involved in genes related to the cell cycle, oocyte meiosis, and inflammatory bowel diseases as well as downregulated microRNAs associated with endocrine resistance, insulin resistance, AGE–RAGE hypersensitivity, and focal adhesion [20].

Defective lymphatic vascularization, which is also considered a contributing factor in other conditions, such as obesity, inflammatory bowel diseases, and neurological disorders, may help explain the presence of musculoskeletal problems (53%), gynecological disorders (27%), anxiety/depression (47%), and gastrointestinal disorders (27%) in patients with lipedema [21].

Similar clinical features were observed in a study involving 77 patients with fibromyalgia and 112 with lipedema [22], and another study of 100 patients over the age of 18 who met the diagnostic criteria for fibromyalgia found lipedema in 50% of cases [23]. This comorbidity may play a role in the occurrence of depression and anxiety, negatively affecting quality of life [24]. In a study of 360 women with lower limb lipedema, compared with the general population, there was a higher prevalence of chronic autoimmune thyroiditis and polycystic ovary syndrome. More than 50% of patients had allergies, and mood disorders, gastrointestinal issues (32.9%), urinary incontinence (25.8%), a history of previous fractures (29%), sprains or dislocations in 20% of cases, and hypermobility in 33% of patients were also reported [25].

This work examined the available evidence on the correlation between connective tissue alterations, HSD, associated pathologies, and symptoms reported in preliminary data from a patient questionnaire, proposing an integrated approach to diagnosis and therapeutic management. Given the documented connective tissue abnormalities and the high prevalence of comorbidities reported in the literature, this study aimed to explore the presence of signs and symptoms associated with HSD in patients with lipedema and lymphedema, and to compare the findings between these two groups.

## 2. Materials and Methods

This research employed a cross-sectional observational design to explore potential associations between lipedema, hypermobility spectrum disorders (HSDs), and connective tissue-related features in a real-world population. Data were collected through an anonymous online survey (Google Forms, Google LLC, Mountain View, CA, USA) distributed between January and March 2025.

The dependent variables included connective tissue manifestations, while the independent variables comprised diagnostic group (lipedema vs. lymphedema) and demographic factors. Participants were explicitly asked whether they had received a clinical diagnosis of lipedema or lymphedema from a healthcare professional. Respondents who did not answer this question or reported uncertainty were excluded from the analysis. Although no external verification (e.g., medical records or imaging) was performed, this approach ensured that only individuals self-reporting a clinical diagnosis were included.

It should be noted that, to date, the diagnosis of lipedema remains clinical, as no unequivocal biomarkers or imaging criteria are universally accepted. Therefore, self-reported diagnostic status was considered acceptable for this exploratory design.

Despite overlapping clinical features such as swelling, fibrosis, and pain, lipedema and lymphedema differ in etiology and connective tissue involvement. Therefore, patients with lymphedema were selected as a comparison group to evaluate shared and distinct pathophysiological patterns, particularly regarding connective tissue and hypermobility-related features. Given the exploratory cross-sectional design and the absence of previous comparable datasets, an a priori sample size calculation was not feasible. Therefore, all consecutive eligible respondents who completed the questionnaire during the recruitment period were included. The final sample size (lipedema = 670; lymphedema = 46) was deemed adequate to provide stable proportion estimates and to explore clinically relevant between-group differences. In the lipedema group, 5.4% of respondents reported being diagnosed with lipolymphedema (coexistence of lipedema and secondary lymphatic insufficiency) [3]. The average age of participants was 40.81 years in the lipedema group and 45.95 years in the lymphedema group. The questionnaire included questions related to common features observed in individuals with hypermobility spectrum disorders such as urogenital issues, gastrointestinal difficulties, musculoskeletal symptoms, movement disorders, and vascular abnormalities. It also collected information on symptoms reported during both childhood and adulthood. Participants were recruited through social media platforms. Responses were collected anonymously. Data analysis was purely descriptive. Frequency distributions and percentages were automatically generated by Google Forms through its built-in summary tools. No additional statistical tests or inferential analyses were performed, as the aim of the study was exploratory, focusing on identifying patterns and associations rather than testing hypotheses. Results are presented as absolute numbers and proportions for each variable.

## 3. Results

**Figure 1:**
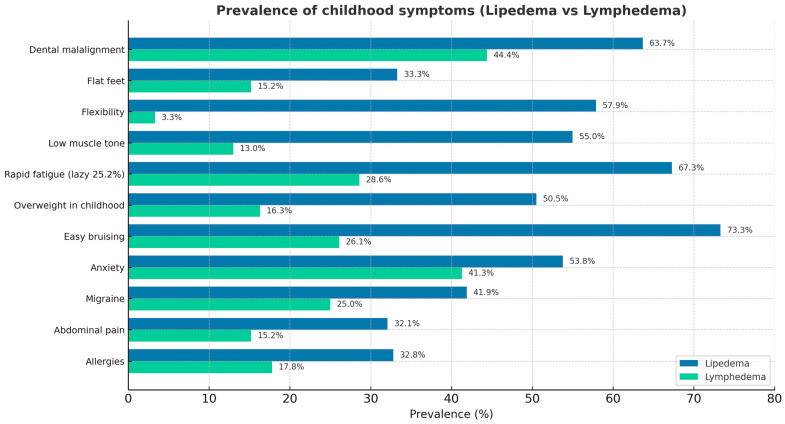
Childhood symptoms in patients with lipedema and lymphedema.

**Figure 2:**
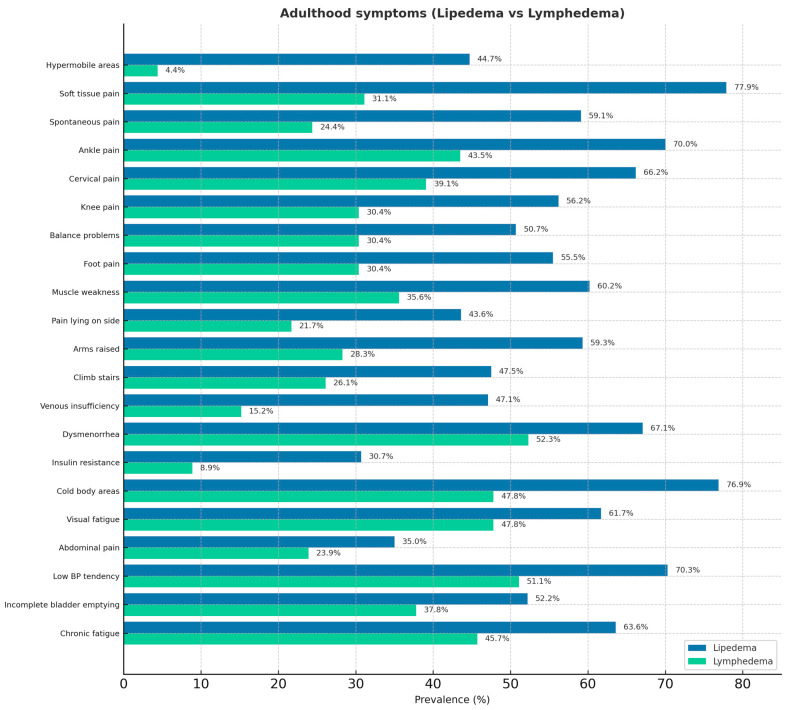
Adult symptoms in patients with lipedema and lymphedema.

The questionnaire results are reported in Figure 1 (childhood age symptoms) and Figure 2 (adult age symptoms). Regarding the shared pathophysiological features of lipedema and HSD, 44% of patients with lipedema reported having hypermobile areas of the body, and approximately 60% recalled being hypermobile children. Reduced muscle tone, reported in 55% of cases, and easy fatigue after physical activity, reported by 70% of patients, are typical symptoms of hypermobility spectrum disorders in childhood. In addition, twenty-six percent of patients with lipedema reported experiencing frequent pain in the legs, knees, back, and cervical spine during childhood. Additionally, 8.79% reported frequent abdominal pain, and 5.66% experienced headaches. Among patients with lymphedema, 12.7% reported pain. In adulthood, 24.4% of patients with lipedema reported thyroid problems (including nodules, hypothyroidism, thyroiditis, or thyroidectomy) compared with 14.89% of patients with lymphedema.

## 4. Discussion

From our analysis of the survey results, the first notable finding was that 44% of patients with lipedema reported having hypermobile areas of the body, and approximately 60% were hypermobile children. These percentages, consistent with data reported in the literature by Torre (2017) [15], prompt consideration of the role of fibrosis in the progressive reduction in mobility over time and the possible under-recognition of hypermobility. Recent studies on HSD increasingly support a multisystemic perspective, highlighting that joint hypermobility alone, traditionally assessed by the Beighton score, is insufficient to capture the full range of extra-articular manifestations. In academic and clinical contexts, new diagnostic criteria are currently under discussion to better reflect this complexity. In patients with lipedema, fibrosis and adipose tissue proliferation may limit the articular range of motion, leading to the underestimation of hypermobility when using the Beighton scale. However, these patients may still exhibit systemic features of connective tissue laxity, suggesting a shared pathophysiological substrate. This overlap implies that comorbidities may stem from underlying connective tissue dysfunction, and that diagnostic and therapeutic approaches should account for these connective tissue traits rather than relying solely on joint mobility scores. The second significant finding is childhood overweight in 50% of patients, in a condition where disease progression is strongly linked to an increase in body fat percentage (Felmerer 2020) [26]. Muscle tone reduction (55% of cases) and easy fatigue after physical activity (70%) are typical symptoms of HSD in childhood. It is known that altered proprioception and interoception are associated with joint instability and play a role in the development of anxiety disorders, which have been reported in the literature as comorbidities of lipedema [15]. Early recognition of these alterations and subsequent therapeutic interventions aimed at improving body awareness and symptom management could have a crucial impact on the patients’ psychological and physical health. In adulthood, the presence of joint pain in various regions (70% ankles, 56% knees, 66% cervical spine, 46% sacrum, 55% feet) highlights the need to shift the understanding of the condition from a localized disorder to a systemic one. This also calls for increased attention—previously focused primarily on fat, extracellular matrix, and the vascular system—toward the articular and fascial connective tissue, especially considering the loss of mobility and autonomy, which represents the most disabling aspect of the disease in advanced stages. The presence of abdominal pain in 30% of pediatric patients and digestive difficulties in 50% of adults leads us to consider gastrointestinal involvement through connective tissue, similar to HSD.

## 5. Limitations

This study presents several limitations that should be acknowledged. First, the cross-sectional design precludes any causal inference regarding the observed associations between connective tissue manifestations and diagnostic group. Second, data were obtained through a self-administered online questionnaire, which may be subject to recall and perception bias. Responses were based on patient-reported information rather than clinical examination. Third, diagnostic confirmation was self-reported, so a degree of misclassification bias cannot be excluded. Fourth, the online and voluntary recruitment strategy may have introduced selection bias, potentially favoring respondents with higher symptom awareness or more severe clinical presentations. Fifth, the absence of a healthy control group limited the ability to define how connective tissue manifestations differ from the general population. Sixth, data analysis was primarily descriptive without advanced inferential statistics; therefore, statistical significance and confounder adjustment were not assessed. Finally, the sample was composed predominantly of Italian respondents, which may limit the generalizability of the findings to other populations or healthcare systems.

An additional limitation concerns the use of a non-validated questionnaire, which may have introduced potential validity bias; further studies are warranted to establish its formal validation.

Nevertheless, while it is true that the study lacks full academic solidity, it must be considered within a research area where robust academic studies are still scarce. By collecting and systematizing the lived experiences of patients, an aspect often underestimated in traditional research, this work introduces an innovative perspective that may help reshape current paradigms and guide future clinically based investigations.

## 6. Conclusions

This cross-sectional study identified distinctive connective tissue and hypermobility-related features differentiating lipedema from lymphedema. Lipedema respondents more frequently reported hypermobile areas, joint pain, perceived stiffness, and multisystem manifestations, whereas lymphedema respondents predominantly presented with lymphatic-related symptoms and rarely reported childhood-onset manifestations. In the lipedema group, the involvement of connective tissue structures, particularly tendons and ligaments, was clearly evident from the analysis of patient-reported data. The collected data suggest that the consequences of connective tissue laxity may represent a significant factor in the lipedema pathogenetic process, causing organ manifestations with systemic impact on the gastrointestinal, vascular, musculoskeletal, urogynecological, and psychological systems. These findings may assist clinicians in distinguishing between the two conditions and inform targeted, multidisciplinary management strategies. Future studies with validated instruments and objective diagnostic criteria are recommended to confirm these observations

## 7. Final Considerations

The broad spectrum of organs affected by problems in lipedema explains the significant degree of physical and psychological suffering often seen in patients with lipedema. Significantly, these organs are often not characterized by particularly evident lipid accumulation. This may indicate that, rather than a disease of the subcutaneous adipose tissue, lipedema should be described as a disease of the connective tissue in its various forms. This appears consistent with the description of a microangiopathy by Michelini (2025) [5] which, however, may cause more noticeable effects when other factors, such as lymphatic or blood stasis, are present. Emerging observations by Aday (2024) indicate that fibro-adipose accumulation might not be limited to the lower limbs and could extend to other regions, especially the upper limb [18]. In these areas, adipose tissue appears to present a characteristic stromal conformation that was clearly visible in both imaging by Conti et al. (2024) [27] and histology (Sbarbati, 2024) [28]. However, the question remains as to whether other organ pathologies present outside the subcutaneous tissue in patients with lipedema should be considered simple comorbidities based on a common genetic diathesis or rather part of a single pathogenic process. These aspects are obviously not negligible as they could be taken into account in establishing an effective therapeutic strategy. The progressive development of the manifestations seems to indicate involvement of the connective tissue’s regenerative mechanisms. Indeed, structures that determine hypermotility such as tendons, joint capsules, and ligaments, which develop in the first years of life, appear to already be involved in childhood. The subcutaneous structures of the lower limb appear to be involved later and progressively, likely in relation to the onset of local (e.g., lymphatic stasis) or general (hormone) conditions. Clearly, the dramatic changes in the subcutaneous tissue, which can reach enormous proportions, have drawn attention to the most obvious features. However, the severity of hypermobility, intestinal, and thyroid dysfunction seems to indicate a functional disorder of the connective tissue stroma that manifests itself in variable ways depending on the organ involved. Furthermore, evidence supporting a systemic nature is growing. Among these, we can mention the existence of specific genetic patterns (Michelini, 2020) [1] and the presence of microangiopathy even in apparently healthy areas, typically unaffected by lipedema (Michelini, 2025) [5]. Joint hyperlaxity is certainly not specific to lipedema and is present in many other pathological conditions, starting with Marfan and EDS. In this latter condition, we have found that the hyperlaxity is linked to the specific alteration of fibroblasts (Chiarelli, 2019) [29]. It would be interesting to test whether similar findings are also present in patients with lipedema and hyperlaxity. Diffuse alterations in the connective tissue have also been recently described in autism spectrum disorders (Veronese, 2023) [30].

The growing evidence of clinical overlap between lipedema and HSD calls for a revision of the diagnostic and therapeutic approach. Early recognition of HSD features in children with a family history of lipedema, especially those presenting with multisystemic symptoms, may represent the first step in prevention, significantly improving the quality of life and guiding patients toward more appropriate care pathways. Multidisciplinary collaboration among specialists is necessary, including rheumatologists, physiatrists, nutritionists, physical therapists, and other allied health professionals, along with the adoption of shared assessment tools and clinical decision-making algorithms. Finally, further studies are required to clarify the relationship between these two conditions and to develop evidence-based therapeutic protocols.

## Data Availability

The data presented in this study are available on request from the corresponding author.
